# Fluconazole-Resistant *Candida glabrata* Bloodstream Isolates, South Korea, 2008–2018

**DOI:** 10.3201/eid2703.203482

**Published:** 2021-03

**Authors:** Eun Jeong Won, Min Ji Choi, Mi-Na Kim, Dongeun Yong, Wee Gyo Lee, Young Uh, Taek Soo Kim, Seung Ah Byeon, Seung Yeob Lee, Soo Hyun Kim, Jong Hee Shin

**Affiliations:** Chonnam National University Medical School, Gwangju, South Korea (E.J. Won, M.J. Choi, S.A. Byeon, S.Y. Lee, S.H. Kim, J.H. Shin);; Asan Medical Center, University of Ulsan College of Medicine, Seoul, South Korea (M.-N. Kim);; Yonsei University College of Medicine, Seoul (D. Yong);; Ajou University School of Medicine, Suwon, South Korea (W.G. Lee);; Yonsei University Wonju College of Medicine, Wonju, South Korea (Y. Uh);; Seoul National University College of Medicine, Seoul (T.S. Kim)

**Keywords:** Candida glabrata, fungal infections, fluconazole resistance, mortality, PDR1, candidemia, antimicrobial resistance, South Korea, fungi

## Abstract

We investigated the clinical outcomes and molecular mechanisms of fluconazole-resistant (FR) *Candida glabrata* bloodstream infections. Among 1,158 isolates collected during multicenter studies in South Korea during 2008–2018, 5.7% were FR. For 64 patients with FR bloodstream infection isolates, the 30-day mortality rate was 60.9% and the 90-day mortality rate 78.2%; these rates were significantly higher than in patients with fluconazole-susceptible dose-dependent isolates (30-day mortality rate 36.4%, 90-day mortality rate 43.8%; p<0.05). For patients with FR isolates, appropriate antifungal therapy was the only independent protective factor associated with 30-day (hazard ratio 0.304) and 90-day (hazard ratio 0.310) mortality. Sequencing of pleiotropic drug-resistance transcription factor revealed that 1–2 additional Pdr1p amino acid substitutions (except genotype-specific Pdr1p amino acid substitutions) occurred in 98.5% of FR isolates but in only 0.9% of fluconazole-susceptible dose-dependent isolates. These results highlight the high mortality rate of patients infected with FR *C*. *glabrata* BSI isolates harboring Pdr1p mutations.

## Abstract 2

Nearly all isolates harbored Pdr1p mutations and were associated with a high mortality rate.

*Candida glabrata* is a commensal yeast in the human gut, genitourinary tract, or oral cavity; however, it can cause serious bloodstream infections (BSIs) that result in substantial illness and death (*1*). Unlike other common *Candida* species, *C. glabrata* exhibits intrinsically low susceptibility to azole drugs, especially fluconazole, and rapidly acquires antifungal resistance in response to azole or echinocandin exposure (*1*–*3*). Although the incidence of echinocandin- and multidrug-resistant (MDR) *C. glabrata* BSIs is low, fluconazole resistant (FR) *C. glabrata* BSI isolates have been increasingly reported worldwide, typically at rates of 2.6%–10.6%, although these rates can reach 17% (*4*–*6*). Fluconazole resistance in *C. glabrata* is of particular concern because of the increased incidence of BSIs caused by this species in various locations worldwide (*1*,*4*,*5*). Acquired azole resistance in *C. glabrata* is most commonly mediated by overexpression of the drug-efflux transporter genes *CgCDR1*, *CgCDR2*, and *CgSNQ2* through a gain-of-function (GOF) mutation in the transcription factor pleiotropic drug-resistance (*PDR1*) (*2*,*7*,*8*), although other mechanisms might contribute (*9*–*11*).

*PDR1* mutations in *C. glabrata* associated with azole resistance have been shown to cause hypervirulence in a mouse model of systemic candidiasis, suggesting the need for careful monitoring of FR *C. glabrata* BSI isolates and their *PDR1* mutations (*7*,*12*). To date, little substantial research has been conducted on *PDR1* mutation incidence among FR *C. glabrata* BSI isolates from multicenter surveillance cultures or on mortality rates of patients infected with these *PDR1* mutants. This deficit might be attributable to Pdr1p amino acid substitutions (AAS) found in FR and fluconazole-susceptible dose-dependent (F-SDD) isolates (*7*,*13*,*14*), which can impede determination of whether specific Pdr1p AAS result in fluconazole resistance. Therefore, the aim of this study was to investigate the clinical outcomes, molecular mechanisms, and genotypes associated with antifungal-resistant BSI isolates of *C. glabrata* collected during multicenter studies in South Korea during an 11-year period (2008–2018). We focused on the mortality rates of patients infected with FR *C. glabrata* BSI isolates harboring the Pdr1p mutation.

## Materials and Methods

### Microorganisms and Antifungal Susceptibility Testing

A total of 1,158 BSI isolates of *C. glabrata* were collected from 19 university hospitals in South Korea during January 2008–December 2018 (Appendix Table 1). All isolates were collected from routine blood cultures by using methods that varied among laboratories; only the first isolate from each patient was included. The hospitals participating in this laboratory-based nationwide multicenter surveillance system differed each year. All *C. glabrata* isolates were submitted to Chonnam National University Hospital (Gwangju, South Korea) for testing. Species identification was based on matrix-assisted laser desorption/ionization time-of-flight mass spectrometry (Biotyper; Bruker Daltonics, https://www.bruker.com) with library version 4.0, or sequencing of the D1/D2 domains of the 26S rRNA gene, to differentiate *C. glabrata* from cryptic species (*C. nivariensis* and *C. bracarensis*) within the *C. glabrata* complex (*15*). In vitro testing of susceptibility to fluconazole, micafungin, caspofungin, voriconazole, and amphotericin B was performed for all isolates according to the Clinical and Laboratory Standards Institute broth microdilution method (*16*). MICs were determined after 24 hours of incubation. Two reference strains, *Candida parapsilosis* ATCC 22019 and *Candida krusei* ATCC 6258, were included in each antifungal susceptibility test as quality-control isolates. The MIC interpretive criteria included species-specific Clinical and Laboratory Standards Institute clinical breakpoints for fluconazole, micafungin, and caspofungin (*17*), as well as epidemiologic cutoff values (ECVs) for voriconazole and amphotericin B (*18*). Echinocandin resistance was confirmed through DNA sequence analysis of *FKS* genes to identify resistance hot-spot mutations in *FKS1* and *FKS2* (*19*). Multidrug resistance was defined as resistance to both fluconazole and echinocandins (*2*).

### Clinical Characteristics

Candidemia was defined as the isolation of *Candida* from >1 blood culture (*20*), and cases with invasive candidiasis without candidemia or colonization were excluded. All demographic characteristics and clinical conditions potentially related to candidemia mortality rates at the time of candidemia onset were investigated (*21*–*23*). Previous use of antifungal agents was defined as administration within 3 months before the onset of candidemia. A lack of antifungal therapy was deﬁned as no antifungal therapy or treatment with antifungals for <3 days; appropriate antifungal therapy was defined as the administration of >1 in vitro–active antifungal (according to the susceptibility pattern of the isolate) for >72 hours (*23*,*24*). Therapeutic failure was defined as either persistence of *Candida* in the bloodstream despite >72 hours of antifungal therapy or development of breakthrough fungemia during treatment with the indicated antifungal agents for >72 hours (*23*,*24*). All-cause mortality rates were assessed at 30 and 90 days after the first positive blood culture result. Mortality rates also were analyzed for patients with candidemia who were infected with 297 SDD isolates of *C. glabrata* as controls. This study was approved by the Institutional Review Board of Chonnam National University Hospital (approval no. CNUH-2020-117).

### Multilocus Sequence Typing and Molecular Mechanisms

Multilocus sequence typing (MLST) and *Pdr1* sequencing were performed for all antifungal-resistant isolates of *C. glabrata* and for 212 F-SDD control isolates by using methods described previously (*14*,*21*,*25*). *Pdr1* sequences of each isolate were compared and analyzed on the basis of the reference *Pdr1* sequence of *C. glabrata* (GenBank accession no. FJ550269) (*14*). The *Fks1* and *Fks2* sequences of 79 isolates that exhibited full or intermediate resistance to micafungin (MIC >0.12 mg/L) or caspofungin (MIC >0.25 mg/L) were compared with those of *C. glabrata* (GenBank reference sequence nos. FKS1 XM_446406 and FKS2 XM_448401) (*14*). The expression levels of *CgCDR1*, *CgCDR2*, and *CgSNQ2* were evaluated for 30 FR isolates of *C. glabrata* harboring FR-specific Pdr AAS and for 65 F-SDD control isolates without FR-specific Pdr AAS, as described previously (*26*,*27*). The cycle threshold (C_t_) of each gene was normalized to that of *URA3* to determine the ΔC_t_ value. For all isolates, relative gene expression (ΔΔC_T_) was reported as fold change calculated as the mean normalized expression level relative to that of *C*. *glabrata* ATCC 90030 (fluconazole MIC 8 mg/L, set as 1.0).

### Statistical Analysis

Quantitative variables are expressed as means with standard deviations, whereas categorical variables are expressed as counts and percentages. Categorical variables were compared by using the χ^2^ test or Fisher exact test, Student *t*–test or the Mann–Whitney U test to compare quantitative variables, as appropriate. Cox proportional hazards models were used to evaluate potential risk factors for 30- and 90-day mortality rates by calculating the hazard ratio (HR). The Kaplan–Meier and log–rank (Mantel–Cox) tests were used to calculate the 30- and 90-day survival probabilities in subgroup analyses. All data were analyzed by using SPSS Statistics 26.0 (IBM, https://www.ibm.com). Statistical significance was determined at a level of p<0.05.

## Results

### Incidence of Antifungal Resistance

The annual proportion of *C. glabrata* BSI isolates among all *Candida* BSI isolates increased from 11.7% to 23.9% (mean 18.6%) during the study period (Table 1). The rate of fluconazole resistance (MIC >64 mg/L) increased from 0% (0/68 isolates) to 8.3% (14/168 isolates) during the study period. Among the 1,158 BSI isolates of *C. glabrata*, 66 (5.7%) were resistant to fluconazole, 16 (1.4%) were resistant to echinocandin, and 6 (0.5%) were resistant to multiple drugs. Of the 16 echinocandin-resistant isolates, 6 (37.5%) were also resistant to fluconazole; thus, these isolates were MDR. Isolates of echinocandin-resistant and MDR *C. glabrata* were initially found in 2013 and then annually from 2016 to 2018. Resistance to amphotericin B (MIC >2 mg/L) was not detected in any isolate, but 79 (6.8%) isolates had voriconazole MICs that exceeded the ECV (0.25 mg/L). All 64 FR isolates were associated with a voriconazole MIC >0.5 mg/L.

**Table 1:** Incidence of antifungal resistance in *Candida glabrata* BSI isolates, based on cultures collected during a multicenter surveillance study, South Korea, 2008–2018*

Study year	No. participating hospitals†	% *C. glabrata* of all *Candida* BSI isolates	No. BSI isolates of *C. glabrata* tested	No. (%) *C. glabrata* BSI isolates‡		
				Fluconazole resistance	Echinocandin resistance§	Multidrug resistance¶
2008	13	11.7	68	0	0	0
2009	8	16.0	67	4 (6.0)	0	0
2010	8	16.8	60	4 (6.7)	0	0
2011	10	16.0	85	4 (4.7)	0	0
2012	11	17.0	108	3 (2.8)	0	0
2013	7	16.9	73	4 (5.5)	1 (1.4)	1 (1.4)
2014	7	22.1	123	11 (8.9)	0	0
2015	10	17.2	110	5 (4.5)	3 (2.7)	0
2016	10	21.2	123	4 (3.3)	4 (3.3)	2 (1.6)
2017	13	21.6	173	13 (7.5)	4 (2.3)	1 (0.6)
2018	13	23.9	168	14 (8.3)	4 (2.4)	2 (1.2)
Total	19	18.6	1158	66 (5.7)	16 (1.4)	6 (0.5)

*BSI, bloodstream infection. †Hospitals participating in this laboratory-based nationwide multicenter surveillance system differed each year. ‡Antifungal susceptibility was determined by using the Clinical and Laboratory Standards Institute M27–4ED broth microdilution method (*16*). Interpretive categories of resistance were determined by using Clinical and Laboratory Standards Institute document M60-ED (*17*). We deposited 76 antifungal-resistant isolates of *C. glabrata* in the Korea Collection for Type Culture (KCTC; Jeongeup-si, Korea), including those showing resistance to fluconazole alone (60 isolates, KCTC nos. 37113–37172), echinocandin alone (10 isolates, KCTC nos. 37176–37185), and both fluconazole and echinocandin (6 multidrug-resistant isolates, KCTC nos. 37110–37112, 37173–37175). All 76 isolates were identified as *C. glabrata* by sequence analysis using the D1/D2 domain (GenBank accession nos. MW349716–90 and MW351777). §Echinocandin resistance was confirmed by the identification of resistance hot-spot mutations in *FKS1* and *FKS2* in isolates that exhibited full or intermediate resistance to micafungin (MIC >0.12 mg/L) or caspofungin MIC (>0.25 mg/L). ¶Multidrug resistance was defined as resistance to both fluconazole and echinocandins.

### Mortality Rate of FR *Candida glabrata* BSIs

The mortality rate for 64 patients with FR *C. glabrata* BSI isolates was 60.9% at 30 days (Appendix Table 2). Univariate Cox regression analyses revealed that a high Charlson comorbidity index (p = 0.051), liver disease (p *=* 0.015), intensive-care unit admission (p *=* 0.071), severe sepsis (p *=* 0.039), lack of antifungal therapy (p<0.001), azole monotherapy (p *=* 0.005), any combination antifungal therapy (p *=* 0.014), and appropriate antifungal therapy (p = 0.001) were associated with the 30-day mortality rate. The 30-day mortality rates were 88.9% (8/9) in patients with azole monotherapy, 69.2% (9/13) in patients with echinocandin monotherapy, 70% (7/10) in patients with amphotericin B monotherapy, 36.4% (8/22) in patients with combination antifungal therapy, 90% (18/20) in patients with inadequate antifungal therapy, and 47.7% (21/44) in patients with appropriate antifungal therapy. Patients treated with azole monotherapy or inadequate antifungal therapy showed significantly higher 30-day mortality rates than those receiving combination therapy or appropriate antifungal therapy (all p<0.05). In multivariate Cox regression analysis, no independent risk factors for 30-day mortality were identified, but appropriate antifungal therapy (HR 0.304 [95% CI 0.134–0.689]; p = 0.004) was independently protective with respect to 30-day mortality. The mortality rate for 64 patients with FR *C. glabrata* BSI isolates was 78.2% at 90 days; appropriate antifungal therapy (HR 0.31 [95% CI 0.138–0.695]; p = 0.004) was the only protective factor with respect to 90-day mortality (Appendix Table 3). Kaplan–Meier survival analysis showed that the mortality dynamics of the FR group (64 patients) decreased during the study period, whereas the F-SDD group (297 patients) exhibited a plateau period of decreasing cumulative survival from 30 to 90 days, which was similar in each of the 4 years of the study period (Figure). The median survival of patients with FR *C. glabrata* BSI was significantly shorter than that of patients with F-SDD *C. glabrata* (17 days for FR vs. 90 days for F-SDD; p<0.001 by log-rank test).

**Figure:**
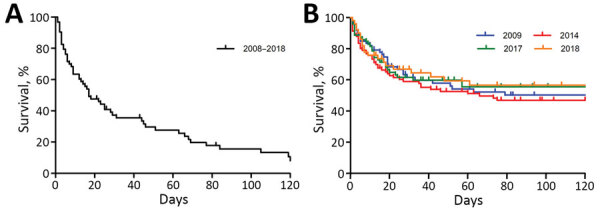
Kaplan–Meier and log-rank (Mantel–Cox) pairwise analyses of survival of patients with *Candida glabrata* candidemia, based on patient data and cultures collected during a multicenter surveillance study, South Korea, 2008–2018. A) Cumulative survival curves of 64 patients infected with fluconazole-resistant (FR) bloodstream infection (BSI) isolates. The cumulative mortality rates of 64 patients infected with FR *C. glabrata* BSIs increased over time (day 7 [29.7%], day 30 [60.9%], day 60 [68.8%], and day 90 [78.1%]). B) Cumulative survival curves of patients infected with fluconazole-susceptible dose-dependent (F-SDD) BSI isolates (297 patients total) in 2009 (75 patients in 6 hospitals), 2014 (97 patients in 7 hospitals), 2017 (75 patients in 9 hospitals), and 2018 (50 patients in 8 hospitals). The 30-day mortality rate of the F-SDD group was 34.7% in 2010, 39.2% in 2014, 37.3% in 2017, and 32.0% in 2018. The cumulative mortality rates of 297 patients infected with F-SDD BSI isolates of *C. glabrata* were found to be 18.5% at day 7 (p = 0.084), 36.4% at day 30 (p = 0.001), 41.8% at day 60 (p<0.001), and 43.8% at day 90 (p<0.001).

### MLST Genotypes and AAS in Pdr1p

MLST revealed that 56.1% (37/66) of FR, 56.3% (9/16) of echinocandin-resistant, and 100% (6/6) of MDR isolates belonged to sequence type (ST) 7. Table 2 lists the sequencing results for *PDR1* and the MLST genotypes for the 66 FR isolates of *C. glabrata*, as well as 212 control F-SDD isolates. In total, 68 types of AAS in Pdr1p were found in the 278 isolates of *C. glabrata* tested. When Pdr1p polymorphisms were compared between >2 isolates in the same ST (257 isolates in 11 STs), excluding 21 STs that were unique to a single isolate, all 50 ST3 isolates harbored the same 3 Pdr1p AAS (P76S, P143T, and D243N), all 8 ST55 isolates harbored E259G, and all 4 ST59 isolates harbored T745A, irrespective of FR. However, these 5 Pdr AAS were not found in any ST7 isolates or any isolates of the other 7 ST groups, each of which contained >2 isolates. Excluding 5 Pdr1 AAS (P76S, P143T, D243N, E259G, and T745A), 1 additional Pdr1p AAS was found in each of 2 F-SDD isolates (0.9%, n = 212); 1 (59 FR isolates) or 2 (6 FR isolates) additional Pdr1p AAS was found in 65/66 (98.5%) FR isolates.

**Table 2:** Pdr1 AAS in 66 FR isolates and 212 F-SDD BSI isolates of *Candida glabrata* and their MLST genotypes, based on cultures collected during a multicenter surveillance study, South Korea, 2008–2018*

MLST genotype	Fluconazole susceptibility	No. isolates tested	No. with echinocandin resistance	No. isolates with 5 Pdr1p AAS found in both FR and F-SDD isolates						No. isolates with additional Pdr1p AAS except for 5 Pdr1p AAS		
				P76S	P143T	D243N	E259G	T745A		1	2	Total
ST7	FR	37	6†							34	3	37
	F-SDD	98	3							0		0
ST3	FR	7	0	7	7	7				6	1	7
	F-SDD	43	1	43	43	43				0		0
ST26	FR	7	0							6		6
	F-SDD	10	1							0		0
ST22	FR	1	0							1		1
	F-SDD	16	1							0		0
ST10	FR	2	0							2		2
	F-SDD	9	0							0		0
ST55	FR	2	0				2			2		2
	F-SDD	6	1				6			1		1
ST2	FR	2	0							2		2
	F-SDD	3	0							0		0
ST6	FR	1	0							1		1
	F-SDD	5	2							0		0
ST59	FR	1	0					1		1		1
	F-SDD	3	1					3		0		0
ST1	FR	2	0							2		2
ST12	F-SDD	2	0							0		0
Other STs‡	FR	4	0				1			2	2	4
	F-SDD	17	0	2	2	2				1		1
Total, no. (%)	FR	66	6	7	7	7	3	1		59	6§	65 (98.5)
	F-SDD	212	10	45	45	45	6	3		2¶		2 (0.9)

*AAS, amino acid substitution; BSI, bloodstream infection; FR, fluconazole-resistant; F-SDD, fluconazole-susceptible dose-dependent; MLST, multilocus sequence typing; ST, sequence type. †All 6 isolates showed multidrug resistance, defined as resistance to both fluconazole and echinocandins. ‡Includes 21 STs that were each unique to a single isolate. §Each of 6 FR isolates harbored 2 additional Pdr1 AAS (E340G/D919Y [ST7], Y556C/F580I [ST7], N132S/G1099S [ST7], F832L/L833V [ST3], G189V/E340G [other ST], and L366P/E555D [other ST]). ¶Two F-SDD isolates harbored additional Pdr1 AAS (V502I [ST55] and R250K [other ST]).

### AAS in Pdr1p Shown in Only FR isolates

Each of the 49 Pdr1p AAS was found alone in 59 FR isolates of *C. glabrata* and their MLST genotypes (Table 3). In 38 (64.4%) isolates, AAS were found in 3 domains of Pdr1p, the inhibition (33.9%), fungal-specific transcription factor (11.9%), and activation (18.6%) domains; AAS were outside the main domains in 21 (35.6%) isolates. Of 49 Pdr1p AAS, 16 were described previously for FR isolates, whereas 33 (67.3%) were newly found in this study. Of these potentially novel Pdr1p AAS, 5 (P327L, G346S, H576Y, T607A, and G788W) were shared by 2 isolates with the same genotype. Among these, 2 AAS (G346S [ST2] and H576Y [ST7]), were shared by 2 isolates from the same hospital in the same year. Quantitative reverse transcription PCR revealed that 30 FR isolates harboring the Pdr mutation exhibited significantly higher mean expression levels of *CgCDR1*, *CgCDR2*, and *CgSNQ2* than 65 control F-SDD isolates (FR vs. F-SDD; 11.5- vs. 1.5-fold for *CgCDR1*, p<0.0001; 43.4- vs. 27.0-fold for *CgCDR2*, p *=* 0.0408; and 4.9- vs. 3.5-fold for *CgSNQ2*, p *=* 0.0174) (Appendix Figure).

**Table 3:** Pdr1 AAS in 59 FR isolates of *Candida glabrata* BSI isolates and their MLST genotypes, based on cultures collected during a multicenter surveillance study, South Korea, 2008–2018*

MLST genotype	No. isolates	Pdr1 AAS (no. isolates)†			
		Inhibition domain	Fungal-specific transcription factor domain	Activation domain	Other regions
ST7	34	P327L (2), G334V (1), **E340G** (1), E340K (1), G346S (1), **L347F** (1), L375P (1), **R376Q** (1), S391L (1)	**H576Y** (2), G583C (1)	**P927S** (1), **G943S** (1), S947L (1), D954N (1), G1088E (1), **Y1106N** (1)	S236N (1), **P258S** (1), **P258L** (1), V260A (1),L280S (1), Y556C (1), E714D (1), T752I (1), **N768D** (1), **R772K** (1), K776E (1), G788W (2), L825P (1), **T885A** (1)
ST26	6	K365E (1), **R376Q** (1), F377I (1), E388Q (1)		N1091D (1)	**S316I** (1)
ST3	6	**L347F** (1)	Y584D (1)	T1080N (1), **Y1106N** (1)	A731E (1), **N764D** (1)
ST1	2		**T607A** (2)		
ST2	2	G346S (2)			
ST10	2	S337F (1), I392M (1)			
ST55	2				F294S (1), **P258S** (1)
ST6	1			**G1079R** (1)	
ST22	1			Y932C (1)	
ST59	1	E369K (1)			
Others	2		L935F (1)		P696L (1)
No. (%) isolates	59	20 (33.9)	7 (11.9)	11 (18.6)	21 (35.6)
No. (%) Pdr1 AAS	49	15 (30.6)	5 (10.2)	10 (20.4)	19 (38.8)

*AAS, amino acid substitutions; BSI, bloodstream infection; FR, fluconazole-resistant; MLST, multilocus sequence typing; ST, sequence type. †Previously reported Pdr1 AAS are shown in bold.

## Discussion

After *C. albicans, C. glabrata* is the most common *Candida* species isolated from BSI in North America and in countries of central and northern Europe (*1*,*4*). *C. glabrata* was the fourth most common BSI-causing *Candida* species in many countries in Asia besides South Korea (*6*,*28*,*29*); however, increasing rates of *C. glabrata* with FR have been reported in China (*30*), and this strain is now the second most common species in South Korea (*31*). In this study, the FR rate of BSI isolates of *C. glabrata* were found to have increased from 0% (0/68) in 2008 to 8.3% (14/168) in 2018. No *C. glabrata* isolate collected during 2008–2012 was resistant to echinocandins, whereas 2%–3% were resistant to echinocandins during 2015–2018. The emergence of echinocandin-resistant BSI isolates of *C. glabrata* in South Korea might reflect the increased use of echinocandin antifungals as the initial option for candidemia after insurance coverage for echinocandins began in 2014 (*32*). Of 16 echinocandin-resistant isolates, 6 (37.5%) were also resistant to fluconazole, indicating multidrug resistance. Overall, our 11-year nationwide surveillance revealed an increasing incidence of *C. glabrata* causing BSI and an increasing propensity for development of antifungal resistance in South Korea, consistent with surveillance data from other countries (*1*,*2*,*4*,*5*,*30*).

Data are scarce regarding the mortality rates for patients with candidemia who are infected with FR *C. glabrata* BSI isolates. The 30-day mortality rates in patients infected with *C. glabrata* BSI isolates are 21.3%–48.6% (*16*,*33*–*37*) but can reach 50%–60% among patients in intensive care units (*38*,*39*). However, few FR *C. glabrata* isolates were included in previous studies. We found that FR BSI isolates of *C. glabrata* in South Korea were associated with significantly higher 30-day (60.9%) and 90-day (78.2%) mortality rates, compared to BSIs caused by F-SDD strains (30-day mortality rate 36.4%, 90-day mortality rate 43.8%). The mortality dynamics of FR isolates indicated a rapid rise in cumulative mortality from 7 to 90 days after BSI onset. This mortality dynamic was distinct from that of patients with F-SDD BSIs, who exhibited a steady curve after 60 days, consistent with previous reports of *C. glabrata* BSIs (*34*,*40*). The median survival of patients with FR *C. glabrata* BSIs (17 days) was also significantly shorter than that of patients with F-SDD *C. glabrata* BSIs (90 days). These findings are consistent with the results in a recent report regarding *C. glabrata* BSIs in South Korea, which showed that a high fluconazole MIC was associated with a poor outcome, although only 5 isolates in that study were FR (*37*).

In this study, MLST revealed that 56.1% of FR and 56.3% of echinocandin-resistant BSI isolates belonged to ST7, which accords with ST7 being the most common MLST genotype (47.8%) in South Korea (*21*). We found that 100% (6/6) of MDR isolates belonged to ST7, which harbored the V239L mutation in the mismatch repair gene (*MSH2*) associated with hypermutability (*21*,*25*). Given that the utility of *MSH2* gene mutations as antifungal-resistance markers remains controversial (*41*,*42*), further surveillance studies are needed. To date, few studies have been conducted on MLST genotype–specific differences in Pdr1p polymorphism among *C. glabrata* BSI isolates. We found that all 50 isolates of ST3 harbored the same Pdr1p AAS (P76S/P143T/D243N), all 7 isolates of ST55 harbored E259G, and all 4 isolates of ST59 harbored T745A, suggesting the presence of MLST genotype–specific Pdr1p AAS. P76S/P143T/D243N in Pdr1p was found to be common in China, Iran, and Australia (*13*,*14*,*43*,*44*), which accords with the high prevalence of ST3 in the study collections. Thus, the results of this study suggest that 5 Pdr1p AAS are MLST genotype–specific; because these AAS were found in both FR and F-SDD isolates, we confirmed that they cannot be responsible for azole resistance.

A single-point mutation in *PDR1* can contribute to azole resistance in *C. glabrata* (*7*,*8*). Our results show that, in FR isolates, AAS are scattered throughout the entire protein without distinct hotspots, as reported previously (*7*,*13*,*41*,*45*). Therefore, determining whether a certain Pdr1p AAS is a GOF mutation is difficult without data from gene editing experiments for all variable regions. A previous study identified 57 FR-specific AAS by comparing azole-susceptible and azole-resistant matched isolates recovered from different clinical specimens (*7*). Furthermore, 91% (74/81) of FR isolates from BSIs or vaginal infections contained a Pdr1 mutation, compared with 5.6% (1/18) of F-SDD isolates (*25*). In our study, we found that 98.5% (65/66) of FR BSI isolates and 0.9% (2/212) of F-SDD BSI isolates harbored an additional 1 or 2 Pdr1p AAS after exclusion of 5 genotype-specific AAS (P76S, P143T D243N, E259G, and T745A). After exclusion of 6 additional FR isolates that harbored 2 Pdr1p AAS (because determining which of the 2 AAS was critical for fluconazole resistance was difficult to determine), we found 49 Pdr1p AAS that were present alone in 59 FR isolates, strongly suggesting that these AAS were FR-specific. Of the 49 Pdr1p AAS, 16 have been described for FR isolates (*7*,*13*,*14*,*25*,*43*–*46*). In this study, FR isolates exhibited higher mean *CgCDR1*, *CgCDR2*, or *CgSNQ2* expression levels, compared with F-SDD isolates; all FR *C. glabrata* isolates were also resistant to voriconazole (MIC >0.5 mg/L), implying that fluconazole and voriconazole resistance are governed by the same mechanism (i.e., a GOF mutation in the transcription factor for Pdr1p) (*7*,*8*). Overall, our findings demonstrate that most FR BSI isolates of *C. glabrata* in South Korea harbor FR-specific Pdr1p AAS.

The cause of the high mortality rate associated with FR *C. glabrata* BSIs remains unclear. In this study, we focused on FR-specific Pdr1p AAS. *PDR1* mutations are associated with increased virulence of *C. glabrata*, expression of adhesins, and adherence to host epithelial cells (*7*,*12*,*47*,*48*). The fungal loads in the kidney, spleen, and liver were higher in mice infected with the FR Pdr1 mutant of *C. glabrata* than in mice infected with F-SDD isolates (*12*). *C. glabrata* might persist in the body by replicating inside phagocytes, eventually leading to cell lysis, rather than by active escape (the method used by *C. albicans*) (*47*,*49*). This process might partly explain the elevated cumulative mortality rate for patients with Pdr1 mutants. Appropriate antifungal therapy was the only independently associated protective factor, with respect to 30- and 90-day mortality rates, in patients infected with FR *C. glabrata* isolates. In patients who received inadequate antifungal therapy and azole monotherapy, the 30-day mortality rates were 90% (antifungal therapy) and 88.9% (azole monotherapy), which were significantly higher than those of the patients receiving combination therapy (36.4%) or appropriate antifungal therapy (47.7%). Previous antifungal exposure was not an independent risk factor for death among patients with FR isolates, although it was identified in 62.5% (40/64) of patients. Given that previous antifungal exposure is a risk factor for antifungal-resistant *Candida* BSI (*50*), further studies including F-SDD *C. glabrata* BSIs might elucidate the relationship between previous antifungal exposure and death. Taken together, these findings suggest that the high mortality rate associated with FR *C. glabrata* BSIs can be explained by the combination of FR and the virulence of Pdr1 mutants.

The first limitation of our study is that a *Candida* species might develop resistance within a patient during antifungal therapy; such resistance can be identified through serial isolates, but we tested only the first isolate from each patient during 2016–2018. Second, our results did not show that FR, *PDR1* mutants, or previous antifungal exposure were independent risk factors for death in patients with *C*. *glabrata* BSIs. A total of 1,158 nonduplicate BSI isolates of *C. glabrata* from 19 university hospitals in South Korea were obtained during the 11-year study period, and the hospitals participating differed each year; therefore, we could not select an appropriate control group of F-SDD isolates. These limitations were partly overcome in a recent study involving 197 adult patients with *C. glabrata* BSI during January 2010–February 2016 at 7 university hospitals in South Korea. In that study, FR was shown to be associated with the 30-day mortality rate in a multivariate analysis (*37*). Third, only limited numbers of patients infected with F-SDD BSI isolates of *C*. *glabrata* were included in our mortality analysis. Nevertheless, we included a total of 297 patients infected with F-SDD BSI isolates of *C*. *glabrata,* which included all patients with *C*. *glabrata* from the participating hospitals in 2010, 2014, 2017, and 2018. The 30-day mortality rates of patients infected with F-SDD *C. glabrata* isolates were similar among those 4 years (32.0%–39.2%), despite differences in participating hospitals and collection periods; the 30-day mortality rate was similar to those reported in previous studies (*21*,*33*–*37*). 

In conclusion, we demonstrated that nearly all FR BSI isolates of *C. glabrata* in South Korea harbored FR-specific Pdr1p mutations by excluding MLST genotype–specific Pdr1p AASs and that the isolates were associated with higher 30-day (60.9%) and 90-day (78.2%) mortality rates. These results suggest that Pdr1 mutants are associated with a risk for death in such patients. In addition, appropriate antifungal therapy was the only independent protective factor against death in patients with FR isolates. Because of the increasing prevalence of FR BSI isolates of *C. glabrata* worldwide, improved detection and appropriate antifungal treatments are critical.

Appendix Additional information about fluconazole-resistant *Candida glabrata* bloodstream isolates, South Korea, 2008–2018.
