# Supplementary material for: Fluconazole-Resistant Candida glabrata Bloodstream Isolates, South Korea, 2008–2018
Source: Emerg Infect Dis. 2021 Mar;27(3):779–88. doi: 10.3201/eid2703.203482 (PMC7920659; doi:10.3201/eid2703.203482)
Supplement: Appendix — Additional information about fluconazole-resistant Candida glabrata bloodstream isolates, South Korea, 2008–2018. [file 20-3482-Techapp-s1.pdf]

Article DOI: https://doi.org/10.3201/eid2703.203482

Fluconazole-Resistant *Candida glabrata* Bloodstream Isolates, South Korea, 2008–2018

Appendix

Appendix Table 1. Distribution of *Candida glabrata* bloodstream-infection (BSI) isolates at participating hospitals during the 11-y study period (2008–2018)

Hospital (no. of hospital beds)	No. (fluconazole-resistant No.) of *C. glabrata* BSI isolates by year*											
	2008	2009	2010	2011	2012	2013	2014	2015	2016	2017	2018	Total
A (2,680)	5 (0)	20 (0)	21 (0)	25 (1)	27 (2)	23 (2)	45 (4)	39 (3)	50 (2)	43 (1)	59 (2)	357 (15)
B (2,437)	19 (0)	9 (1)	13 (3)	11 (0)	21 (0)	17 (0)	33 (5)	1 (0)	23 (2)	34 (7)	41 (6)	222 (26)
C (1,989)	6 (0)											6 (0)
D (1,741)									4 (0)	25 (3)	12 (1)	41 (4)
E (1,453)	2 (0)	9 (0)		14 (2)	12 (0)							37 (2)
F (1,376)	8 (0)				2 (0)							10 (0)
G (1,300)	8 (0)	10 (0)	3 (1)	6 (0)	5 (0)			10 (0)	5 (0)			47 (1)
H (1,224)	5 (0)	6 (0)	6 (0)	5 (0)	9 (0)	5 (2)	9 (2)	13 (0)				58 (4)
I (1,092)	1 (0)		6 (0)	5 (0)	5 (0)	6 (0)	8 (0)	5 (0)	10 (0)	4 (0)	8 (0)	58 (0)
J (1,084)	11 (0)	5 (0)	8 (0)	8 (0)	14 (0)	17 (0)	16 (0)	21 (0)	10 (0)	20 (1)	18 (2)	148 (3)
K (919)	2 (0)				5 (0)	4 (0)		2 (0)				13 (0)
L (903)									3 (0)	5 (0)	3 (1)	11 (1)
M (880)										6 (1)	5 (1)	11 (2)
N (866)				8 (0)	1 (0)		10 (0)	13 (2)	8 (0)	14 (0)	8 (1)	62 (3)
O (840)	1 (0)	1 (0)	1 (0)	2 (0)							0 (0)	5 (0)
P (814)									4 (0)	6 (0)	9 (0)	19 (0)
Q (803)									3 (0)	2 (0)	1 (0)	6 (0)
R (745)									3 (0)	12 (0)	1 (0)	16 (0)
S (705)		7 (3)	2 (0)	1 (1)	7 (1)	1 (0)	2 (0)	6 (0)		2 (0)	3 (0)	31 (5)
Total	68 (0)	67 (4)	60 (4)	85 (4)	108 (3)	73 (4)	123 (11)	110 (5)	123 (4)	173 (13)	168 (14)	1158 (66)

*Sequential isolates of *C. glabrata* from the same patient were collected, and the first and last isolates from the same patient were tested if available (897 isolates from 694 patients) from 2008 to 2015. During this period, development of acquired resistance to both azole and echinocandin (multidrug resistance) in sequential isolates was detected in only one patient from hospital A in 2013. Only the first isolate was collected and tested from 2016 to 2018 (464 isolates from 464 patients). Overall, 1,158 isolates from 1,158 patients were included in the analysis, to minimize the bias associated with reporting repeated cultures.

Appendix Table 2. Thirty day mortality-associated factors for 64 patients infected with fluconazole resistant *Candida glabrata* bloodstream isolates, analyzed by univariate and multivariate Cox-regression models*

	Value for group[‡]		Univariate analysis		Multivariate analysis[¶]	
Clinical variables	Survival (n = 25; 39.1%)	Death (n = 39; 60.9%)	HR (95% CI)	p value	HR (95% CI)	p value
Demographic information						
Old age (>65 y)	8 (32.0)	16 (41.0)	1.382 (0.730–2.617)	0.321		
Male	11 (44.0)	23 (59.0)	1.417 (0.748–2.682)	0.285		
Underlying disease						
Hematologic malignancies	7 (28.0)	15 (38.5)	1.145 (0.600–2.187)	0.681		
Diabetes mellitus	8 (32.0)	14 (35.9)	1.259 (0.653–2.424)	0.492		
Solid tumor	9 (36.0)	11 (28.2)	0.883 (0.439–1.776)	0.727		
Chronic kidney disease	5 (20.0)	12 (30.8)	1.433 (0.726–2.832)	0.3		
Liver disease	2 (8.0)	13 (33.3)[§]	2.319 (1.178–4.564)	0.015	1.514 (0.642–3.569)	0.343
Connective tissue disease	4 (16.0)	3 (7.7)	0.527 (0.162–1.713)	0.287		
Congestive heart failure	1 (4.0)	3 (7.7)	1.130 (0.347–3.675)	0.84		
Myocardial infarction	2 (8.0)	2 (5.1)	1.080 (0.260–4.492)	0.916		
COPD	2 (8.0)	2 (5.1)	0.838 (0.202–3.481)	0.808		
Hemiplegia	1 (4.0)	3 (7.7)	4.689 (1.379–15.938)	0.013		

Clinical variables	Value for group‡ Survival (n = 25; 39.1%)	Death (n = 39; 60.9%)	Univariate analysis HR (95% CI)	p value	Multivariate analysis¶ HR (95% CI)	p value
Peptic ulcer disease	1 (4.0)	2 (5.1)	1.155 (0.278–4.796)	0.843		
Dementia	1 (4.0)	1 (2.6)	0.946 (0.130–6.902)	0.956		
AIDS	0 (0.0)	1 (2.6)	12.131 (1.417–103.837)	0.023		
Peripheral vascular disease	0 (0.0)	1 (2.6)	5.845 (0.748–45.670)	0.092		
Clinical status at positive culture						
ICU admission	10 (40.0)	26 (66.7)§	1.849 (0.949–3.603)	0.071	1.223 (0.566–2.644)	0.609
ACCI	4.60 ±3.028	6.15 ±3.689§	1.122 (1.021–1.233)	0.017	1.047 (0.927–1.182)	0.458
Candida score	1.52 ±1.046	1.90 ±0.995	1.202 (0.879–1.643)	0.249		
Central venous catheter	21 (84.0)	37 (94.9)	1.905 (0.459–7.914)	0.375		
Total parenteral nutrition	19 (76.0)	30 (76.9)	0.826 (0.392–1.742)	0.616		
Urine catheter	18 (72.0)	29 (74.4)	1.050 (0.511–2.157)	0.894		
Severe sepsis	9 (36.0)	26 (66.7)§	2.018 (1.035–3.937)	0.039	1.535 (0.682–3.453)	0.3
Immunosuppressive therapy	13 (52.0)	17 (43.6)	0.614 (0.325–1.160)	0.133		
Neutropenia	8 (32.0)	14 (35.9)	1.171 (0.608–2.255)	0.637		
Prior surgery (1 mo)	5 (20.0)	6 (15.4)	0.781 (0.327–1.865)	0.578		
Previous use of antifungals	15 (60.0)	25 (64.1)	1.035 (0.538–1.991)	0.919		
Prior azole exposure	14 (56.0)	23 (59.0)	1.068 (0.564–2.022)	0.84		
Prior echinocandins exposure	4 (16.0)	5 (12.8)	0.824 (0.322–2.108)	0.686		
Prior amphotericin B exposure	2 (8.0)	4 (10.3)	0.972 (0.345–2.738)	0.958		
Breakthrough fungemia	6 (24.0)	11 (28.2)	1.082 (0.538–2.176)	0.824		
Antifungal treatment after diagnosis						
Lack of antifungal therapy	1 (4.0)	9 (23.1)§	5.753 (2.496–13.257)	<0.001	2.084 (0.762–5.702)	0.153
Azole monotherapy	1 (4.2)	8 (26.7)§	3.282 (1.444–7.460)	0.005		
Echinocandin monotherapy	4 (18.7)	9 (30.0)	1.596 (0.729–3.493)	0.242		
Amphotericin B monotherapy	3 (30.0)	7 (20.6)	0.871 (0.333–2.278)	0.779		
Combination therapy	14 (58.3)	8 (26.7)§	0.359 (0.159–0.810)	0.014		
Appropriate antifungal therapy†	23 (92.0)	21 (53.8)§	0.232 (0.120–0.449)	<0.001	0.304 (0.134–0.689)	0.004
Therapeutic failure	12 (48.0)	18 (46.2)	0.794 (0.422–1.494)	0.474		
Isolate factor						
Echinocandin resistance (MDR)	2 (8.0)	3 (7.7)	0.879 (0.271–2.856)	0.83		

*COPD, chronic obstructive pulmonary disease; AIDS, acquired immunodeficiency syndrome; ICU, intensive care unit; ACCI, age-adjusted Charlson comorbidity index; MDR, multidrug resistance; HR, hazard ratio; CI, confidence interval.
†Of 64 patients, 44 were included in the analysis; we excluded 20 patients who had received inadequate antifungal therapy (10 patients with no antifungal therapy or therapy duration of <3 d; 10 patients were treated with antifungal agents to which the *Candida* isolate was likely to be resistant [9 with fluconazole and 1 with echinocandin monotherapy for ≥72 h]).
‡ Percentages in parentheses were calculated relative to the total number of patients infected with fluconazole resistant *C. glabrata* bloodstream isolates in each group, with the exceptions of two quantitative variables (ACCI and *Candida* score), which are expressed as means with standard deviations.
§ Statistical significance (p<0.05) between the survival and death groups within a given category.
¶Variables with p<0.1 by univariate analysis were evaluated by multivariate analysis.

Appendix Table 3. Ninety day mortality-associated factors for 64 patients infected with fluconazole resistant *Candida glabrata* bloodstream isolates, analyzed by univariate and multivariate Cox-regression models*

Clinical variables	Value for group† Survival (n = 14; 21.9%)	Death (n = 50; 78.1%)	Univariate analysis HR (95% CI)	p value	Multivariate analysis§ HR (95% CI)	p value
Demographic information						
Old age (>65 y)	3 (21.4)	21 (42.0)	1.384 (0.788–2.432)	0.258		
Male	5 (35.7)	29 (58.0)	1.352 (0.770–2.373)	0.293		
Underlying disease						
Hematologic malignancies	5 (35.7)	17 (34.0)	0.990 (0.550–1.783)	0.974		
Diabetes mellitus	6 (42.9)	16 (32.0)	1.102 (0.607–2.001)	0.749		

	Value for group†		Univariate analysis		Multivariate analysis§	
	Survival	Death				
Clinical variables	(n = 14; 21.9%)	(n = 50; 78.1%)	HR (95% CI)	p value	HR (95% CI)	p value
Solid tumor	3 (21.4)	17 (34.0)	1.087 (0.604–1.957)	0.78		
Chronic kidney disease	3 (21.4)	14 (28.0)	1.322 (0.712–2.454)	0.377		
Liver disease	0 (0.0)	15 (30.0)‡	2.278 (1.228–4.228)	0.009	1.929 (0.793–4.690)	0.147
Connective tissue disease	4 (28.6)	3 (6.0)‡	0.388 (0.120–1.248)	0.112		
Congestive heart failure	1 (7.1)	3 (6.0)	0.843 (0.262–2.712)	0.774		
Myocardial infarction	1 (7.1)	3 (6.0)	1.524 (0.470–4.944)	0.483		
COPD	1 (7.1)	3 (6.0)	0.885 (0.275–2.850)	0.837		
Hemiplegia	1 (7.1)	3 (6.0)	4.689 (1.379–15.938)	0.013		
Peptic ulcer disease	0 (0.0)	3 (6.0)	1.401 (0.434–4.524)	0.573		
Dementia	1 (7.1)	1 (2.0)	0.514 (0.070–3.756)	0.512		
AIDS	0 (0.0)	1 (2.0)	12.131 (1.417–103.837)	0.023		
Peripheral vascular disease	0 (0.0)	1 (2.0)	5.845 (0.748–45.670)	0.092		
Clinical status at positive culture						
ICU admission	3 (21.4)	33 (66.0)‡	2.178 (1.199–3.955)	0.011	1.621 (0.792–3.321)	0.186
ACCI	3.57 ±2.593	6.10 ±3.547‡	1.121 (1.032–1.218)	0.007	1.043 (0.937–1.161)	0.438
Candida score	1.0 ±0.679	1.96 ±1.009‡	1.283 (0.979–1.681)	0.071	1.083 (0.470–2.494)	0.851
Central venous catheter	12 (85.7)	46 (92.0)	1.216 (0.436–3.391)	0.709		
Total parenteral nutrition	10 (71.4)	39 (78.0)	0.885 (0.452–1.729)	0.72		
Urine catheter	9 (64.3)	38 (76.0)	1.249 (0.651–2.398)	0.504		
Severe sepsis	1 (7.1)	34 (68.0)‡	2.567 (1.402–4.698)	0.002	1.716 (0.810–3.635)	0.158
Immunosuppressive therapy	9 (64.3)	21 (42.0)	0.548 (0.311–0.966)	0.038	0.485 (0.231–1.018)	0.056
Neutropenia	5 (35.7)	17 (34.0)	0.993 (0.551–1.787)	0.98		
Prior surgery (1 mo)	2 (14.3)	9 (18.0)	0.793 (0.383–1.641)	0.531		
Previous use of antifungals	9 (64.3)	31 (62.0)	0.989 (0.558–1.753)	0.971		
Prior azole exposure	9 (64.3)	28 (56.0)	0.964 (0.551–1.687)	0.899		
Prior echinocandins exposure	1 (7.1)	8 (16.0)	1.166 (0.546–2.491)	0.692		
Prior amphotericin B exposure	1 (7.1)	5 (10.0)	0.990 (0.393–2.498)	0.984		
Breakthrough fungemia	3 (21.4)	14 (28.0)	1.073 (0.578–1.993)	0.822		
Antifungal treatment after diagnosis						
Lack of antifungal therapy	1 (7.1)	9 (18.0)	5.753 (2.496–13.257)	<0.001	1.374 (0.456–4.141)	0.573
Azole monotherapy	1 (7.7)	8 (19.5)	2.344 (1.073–5.122)	0.033		
Echinocandin monotherapy	2 (15.4)	11 (26.8)	1.624 (0.807–3.268)	0.174		
Amphotericin B monotherapy	3 (30.0)	7 (20.6)	0.772 (0.341–1.751)	0.536		
Combination therapy	7 (53.8)	15 (36.6)	0.550 (0.290–1.044)	0.068		
Appropriate antifungal therapy	12 (85.7)	32 (64.0)	0.303 (0.165–0.555)	<0.001	0.310 (0.138–0.695)	0.004
Therapeutic failure	5 (35.7)	25 (50.0)	0.920 (0.527–1.605)	0.769		
Isolate factors						
Echinocandin resistance (MDR)	0 (0.0)	5 (10.0)	1.177 (0.466–2.971)	0.73		

*COPD, chronic obstructive pulmonary disease; AIDS, acquired immunodeficiency syndrome; ICU, intensive care unit; ACCI, age-adjusted Charlson comorbidity index; MDR, multidrug resistance; HR, hazard ratio; CI, confidence interval.

†Percentages in parentheses were calculated relative to the total number of patients infected with fluconazole resistant *C. glabrata* bloodstream isolates in each group, with the exceptions of two quantitative variables (ACCI and *Candida* score), which are expressed as means with standard deviations.

‡Statistical significance ($p<0.05$) between the survival and death groups within a given category.

§Variables with $p<0.1$ by univariate analysis were evaluated by multivariate analysis.

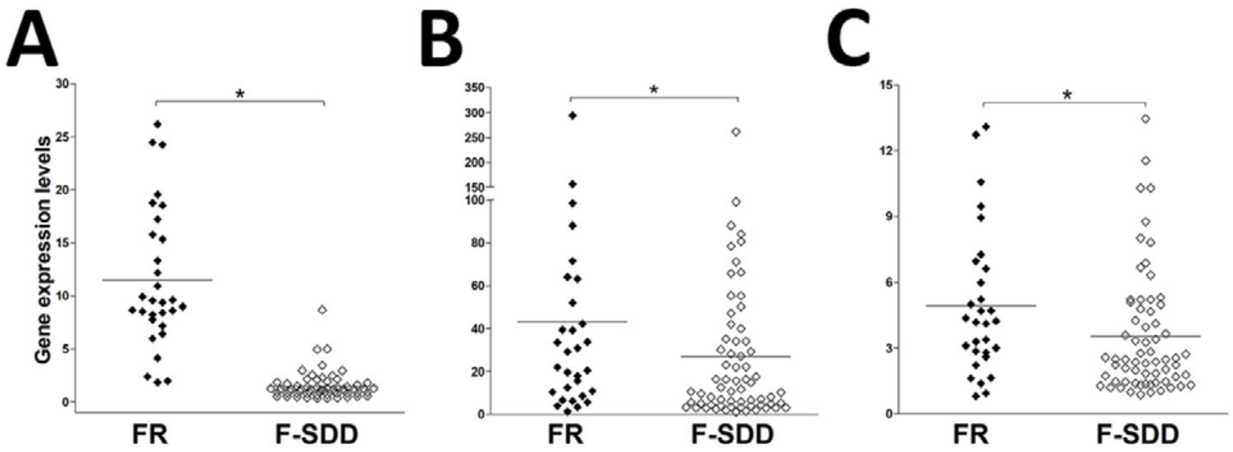

Appendix Figure. Relative expression levels of *CgCDR1*, *CgCDR2*, and *CgSNQ2* genes evaluated after of *C. glabrata* isolates (30 FR isolates harboring the Pdr mutation and 65 control F-SDD isolates) to fluconazole. Expression levels of each gene were calculated relative to the mean normalized expression level of *C*. *glabrata* ATCC 90030 (set as 1.0). Each symbol represents an individual FR (filled diamond) and F-SDD (open diamond) isolate. Horizontal bars indicate mean gene expression levels of each group. *p<0.05.
